# Bilosomal Co-Encapsulated Tamoxifen and Propranolol for Potentiated Anti-Breast Cancer Efficacy: In Vitro and In Vivo Investigation

**DOI:** 10.3390/pharmaceutics17010123

**Published:** 2025-01-17

**Authors:** Toka T. Elebyary, Amal A. Sultan, Sally E. Abu-Risha, Gamal M. El Maghraby, Manna Amin

**Affiliations:** 1 Department of Pharmaceutical Technology, Faculty of pharmacy, Tanta University, Tanta 31527, Egypt; tokaelebyary@gmail.com (T.T.E.); gamal.elmaghraby@pharm.tanta.edu.eg (G.M.E.M.); 2 Educational Hospital, Tanta University, Tanta 31527, Egypt; 3 Department of Pharmaceutics, College of Pharmacy, University of Hafr Al-Batin, Hafr Al-Batin 39911, Saudi Arabia; 4 Department of Pharmacology and Toxicology, Faculty of Pharmacy, Tanta University, Tanta 31527, Egypt; sally.abouresha@pharm.tanta.edu.eg; 5 Faculty of Pharmacy, Alsalam University, Tanta 31527, Egypt; 6 Department of Pharmaceutical Sciences, Faculty of Pharmacy, Umm Al-Qura University, Makkah 21955, Saudi Arabia; maamin@uqu.edu.sa

**Keywords:** tamoxifen, propranolol, bilosomes, niosomes, anti-breast cancer

## Abstract

**Background/Objectives**: Tamoxifen (TAM) is an anti-breast cancer drug suffering from acquired resistance development, prompting cancer relapse. Propranolol (PRO)’s repurposing for cancer therapy has gained interest. This work aimed to investigate combined TAM/PRO therapy for potentiating the anti-breast cancer activity of TAM. The work probed bilosomes versus standard noisome for simultaneous oral and intratumor delivery of TAM and PRO. **Methods**: Bilosomes comprising Span60, cholesterol, and increasing concentrations of bile salts were prepared together with bile salts containing free standard niosomes. The vesicular size and morphology were characterized. The entrapment and release efficiencies of TAM and PRO from the tailored vesicles were determined. The in vivo investigations of anti-tumor activity of TAM with or without PRO employed the solid Ehrlich carcinoma model. **Results:** The vesicles of all fabricated dispersions were spherical and negatively charged, with a size ranging from 104 to 182 nm. The entrapment efficiency depended on the nature of the drug, recording values ranging from 87.5% to 97.8% for TAM and from 31.0% to 46.8% for PRO. Incorporation of bile salts into vesicles increased TAM and PRO release compared to standard niosomes. Oral administration of combined TAM/PRO bilosomes showed a significant reduction in tumor growth volume compared to that recorded following naked drug administration. Histopathological investigations reflected a significant decline in tumor giant cells and mitotic figures, implying the in vivo capability of the TAM/PRO combination to interfere with cancer cell proliferation and persistence. **Conclusions**: The overall results demonstrated the impact of repurposed PRO to enhance the anti-breast cancer activity of TAM when both were co-encapsulated into bilosomes.

## 1. Introduction

The incidence of multidrug resistance to anti-cancer agents is growing with time. Despite the technological advancements in cancer treatments, the issue of drug resistance still presents a major obstacle for most traditional and innovative breast cancer therapies. The efficacy of chemotherapeutic drugs is hindered by multidrug resistance (MDR), inducing relapse and metastasis. Both innate and acquired resistance are implicated in the treatment failure of breast cancer. Numerous mechanisms have been suspected for drug resistance. These include increased drug efflux, improved DNA repair, epigenetic alterations, escape from senescence, tumor microenvironment heterogeneity, and epithelial-to-mesenchymal transition [1]. This requires the development of new anti-cancer agents. This development is complicated by the required lengthy procedures and high budget. For example, the average research and development cost of introducing a single anti-cancer drug to market has been estimated to reach USD 2.7 billion [2]. The newly developed anti-cancer agents (if any) are always marketed at very high prices, which increases the cost of treatment for individual patients. Therefore, searching for a suitable alternative to reduce the financial burden on patients is crucial. This drew the attention of researchers to test the anti-cancer potential of already available non-anti-cancer drugs in a process called drug repurposing [3]. Repurposing of existing drugs for cancer treatment offers a rational and cost-effective approach to provide solutions to these problems. This process is less expensive and less time-consuming, and it can avoid the toxicity and safety determination, which is very expensive [4].

**Figure 1:**
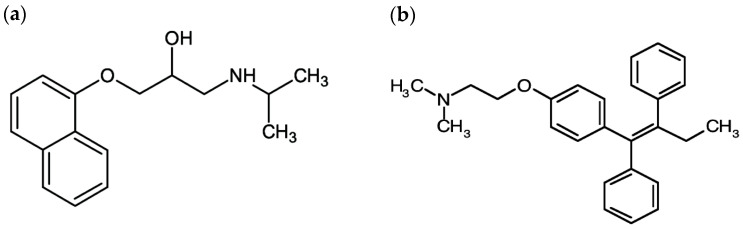
Chemical structure of (**a**) propranolol (C_16_H_21_NO_2_), and (**b**) tamoxifen (C_26_H_29_NO).

Propranolol (PRO) is one such drug that has been highly investigated for repurposing as a possible anti-cancer drug. Propranolol is a non-selective antagonist to the beta-adrenergic receptor (Figure 1a). It is essentially used to treat hypertension [5,6]. Recently, propranolol became a valuable model as a drug with many off-target therapeutic properties, which has led to drug repurposing. Propranolol displays many pharmacodynamic properties, such as the induction of apoptosis and the inhibition of angiogenesis. These effects were reported to explain the efficacy of propranolol in treating infantile hemangiomas [7,8,9]. This highlighted its therapeutic possibilities as an anti-cancer drug, especially in vascular tumors [10]. This, together with its low cost, tolerability, and in vitro ability to prevent tumor progression, make it a good candidate for cancer repurposing in humans [11]. The above-mentioned specifications can dictate a high potential for propranolol to at least potentiate the efficacy of existing anti-cancer agents, which can subsequently allow for the reduction of the dose and side effects of anti-cancer agents. The drug is categorized as class I (highly soluble and highly permeable) according to the biopharmaceutical classification system [12]. Unfortunately, extensive pre-systemic metabolism results in its low and variable oral bioavailability, with the bioavailability ranging from 25 to 35% after oral administration [13].

Tamoxifen (TAM) is the most extensively used drug for the treatment of estrogen receptor (ER)-positive breast cancer (Figure 1b). It functions through competitive inhibition of estrogen from binding to the estrogen receptor, thus stopping the receptor from binding to the estrogen-response element on DNA [14,15]. TAM is employed as long-term prophylactic therapy for post-menopausal women and others who are categorized as high-risk subjects for breast cancer development [16]. Studies have indicated the existence of intrinsic resistance to hormonal therapies, including TAM. In addition, many patients with localized breast cancer and almost 100% of those suffering from advanced breast cancer who show an initial response to TAM therapy exhibit de novo or acquired resistance [17,18]. Combined therapy may positively contribute to solving this problem. Co-administration of TAM with PRO may be beneficial in this case, but the efficacy requires verification. The oral bioavailability of TAM is variable due to poor dissolution and pre-systemic metabolism, with researchers highlighting a role of intestinal efflux transporters [19].

The development of lipid-based nano-systems is claimed to enhance the intestinal permeability of loaded drugs [20]. Lipid-based vesicular systems, like niosomes, liposomes, and bilosomes, are claimed to enhance oral bioavailability through intact vesicular absorption through lymphatics. This can bypass the hepatic metabolism, which is another advantage. Moreover, the absorption will not be dissolution-rate-limited [21,22,23]. These features can provide benefits for hydrophilic, poorly permeable drugs, in addition to those suffering from extensive pre-systemic metabolism. Surfactant-based niosomal vesicles have been reported to provide additional benefits when compared with liposomes. These include the relatively lower cost, good stability, ease of formulation, and scaling up [24,25]. Accordingly, the objective of this work was to investigate surfactant vesicles for simultaneous oral delivery of TAM and PRO. This involved nonionic surfactant-based standard niosomes and bile salts containing niosomes. The latter is termed bilosomes and was previously reported to improve the flexibility, absorption potential, and permeability of conventional niosomes [23]. This study involved in vitro characterization before in vivo investigations using a solid Ehrlich carcinoma mouse model. Intratumor drug administration directly delivers the anti-tumor agents into the tumor, with subsequent concentration increments of the anti-tumor drugs. This would enhance the efficacy and reduce the systemic toxicity [26]. The current study aims to improve the oral delivery of vesicular-loaded combined TAM/PRO therapy. Accordingly, the in vivo efficacy of the selected formulations was assessed following oral administration and compared to the intratumor route.

## 2. Materials and Methods

### 2.1. Materials

TAM was obtained as a gift sample from Pharco Corporation, Borg El Arab, Egypt. PRO was a gift sample from Cairo Pharmaceuticals and Chemical Industry, Cairo, Egypt. Span 60 was obtained from Oxford Lab Fine Chem. LLP, Vasai East, Thane, Maharashtra, India. Bile salts (sodium cholate/sodium deoxycholate 1:1) and cholesterol were acquired from Sigma Aldrich Chemical Co., St. Louis, MO, USA. Hydrochloric acid and ethanol were purchased from El-Nasr Pharmaceutical Chemicals Co., Cairo, Egypt.

### 2.2. Spectrophotometric Quantification of TAM and PRO

Analysis of TAM and PRO was performed using UV spectrophotometry. Calibration graphs were separately constructed by dissolving 100 mg of TAM or PRO in 100 mL of ethanol to prepare stock solutions of 1 mg/mL. The stock solutions were appropriately diluted with ethanol to obtain sequence concentrations of 5, 10, 15, 20, and 25 µg/mL for TAM and 5, 10, 15, 20, 25, 30, and 35 µg/mL for PRO. The absorbances were then measured spectrophotometrically at 237 nm for TAM and 290 nm for PRO. The calibration curves were constructed by plotting absorbance values as a function of concentrations. The assay methods were validated regarding linearity, precision, accuracy, the detection limit, and the quantification limit. Assay validation was performed in compliance with the guidelines of the International Conference on Harmonization. Analytical procedures were validated in order to confirm that the procedure was suitable for its planned use. Validation was carried out to reveal that the result(s) created by specific analytical procedures were accurate and reliable.

### 2.3. Preparation of Standard Niosomes and Bilosomes

The adopted procedure for standard niosomes and bilosomes preparation was previously described in the literature. The tested niosomal and bilosomal dispersions were designed to incorporate a total lipid concentration of 40 mg/mL. The composition of the fabricated vesicles is listed in Table 1. The method is based on preparing standard niosomes and bilosomes from their pro-concentrates [22,23]. Span 60, cholesterol, bile salt (if present), TAM, PRO, and ethanol were heated on a water bath (at 80 °C) until clear liquid formation. An aqueous phase (with a volume equal to that of ethanol) heated up to the same temperature was added with rapid mixing (using a glass rode) while heating until the formation of homogenous dispersion. This pro-concentrate was hydrated gradually with the remaining aqueous phase to obtain the niosomal or bilosomal dispersion. The developed dispersions were prepared to contain 1 mg/mL of tamoxifen or propranolol. The fabricated vesicular dispersions were kept at ambient temperature overnight for swelling prior to down-sizing using bath sonication for 30 min (Bransonic 3510E–MTH, Branson Ultrasonics Corporation, Danbury, CT, USA).

### 2.4. Transmission Electron Microscopy

The morphology of vesicles was determined through TEM (JEM-1400 Plus, Jeol, Tokyo, Japan). Sample preparation involved mounting one drop of the vesicular dispersion on a carbon cupper plate. This was left to dry before staining with uranyl acetate. The plates were then mounted in the holder of the equipment for examination and photomicrography.

### 2.5. Determination of Particle Size and Zeta Potential

The average particle sizes (PSs) with their polydispersity indices (PDIs), as well as zeta potential (ZP) values of the developed vesicular systems, were determined by employing Photon Correlation Spectroscopy (PCS). Each vesicular dispersion was diluted with about a 0.2 μm of filtered distilled water prior to measurement using a dynamic light scattering instrument (Brookhaven Instruments Corp., Holtsville, NY, USA). All measurements were conducted in triplicates.

### 2.6. Determination of Entrapment Efficiency

The entrapment efficiency expressed as the percentage (EE) of tamoxifen and propranolol was measured separately. Entrapment efficiency was determined indirectly by computing the unentrapped drugs in the dispersion medium. From each vesicular dispersion, 3 mL samples were centrifuged at 20,000 rpm for about 90 min at 4 °C using a high-speed centrifuge (SIGMA 3–30 K centrifuge, Osterode am Harz, Lower Saxony, Germany) [27]. The separated supernatant was then decanted and diluted with ethanol before free drug quantification. The entrapment efficiency was computed using the following equation [23]:

%EE = (Total Drug conc. − Free Drug conc.)/(Total Drug conc.) × 100

### 2.7. In Vitro Drug Release

The in vitro release of TAM and PRO was monitored from the tailored vesicular systems and the aqueous drug dispersions (control) utilizing a diffusion strategy. This involved employing Franz cells, where the average diffusional surface area was about 2.27 cm^2^. The used semipermeable membrane was cellulose tubing with an MW cutoff 14,000 (Sigma Aldrich, St. Louis, MO, USA). To ensure a constant pore diameter throughout the release study, the membrane was soaked in distilled water for 24 h for equilibration and swelling before mounting on the Franz cells. The selected molecular weight cutoff was significantly greater than the molecular weight of PRO (259.34 g/mol) and TAM (563.64 g/mol), allowing for free diffusion of both drugs. The receptor compartment was filled with 0.02 N HCL (receptor medium). The donor compartment was loaded with 2 mL of each fabricated niosome and bilosome dispersion incorporating either TAM or PRO. The donor chambers were occluded with aluminum foil. The cells were kept in a thermos-stated water bath retained at 37 °C throughout the whole experiment. At the specified time interval, samples (5 mL each) of the receptor fluid were withdrawn and replaced by fresh receptor media to guarantee a fixed volume. The amount of drug released was computed using UV spectroscopy at 237 and 290 nm for TAM and PRO, respectively, after suitable dilution with the receptor fluid, if necessary. Release profiles were then constructed via plotting the percentage of the cumulative amount of drug released as a function of time. The release efficiency was calculated according to Khan and listed as a percentage [28]. TAM and PRO release from the control aqueous dispersions (1 mg/mL) was similarly investigated. The release profiles were correlated for comparison by employing the similarity factor (f2 value) test. According to the FDA, an f2 value < 50% indicates different release profiles, while those >50 signify similar release behavior [29].

Additionally, the release profiles were fitted to different zero order, first order, and Higuchi kinetic models to determine the kinetics of drug release. The kinetic model providing the highest correlation coefficient value was taken as the model describing the release data.

### 2.8. In Vivo Evaluation of Anti-Tumor Activity

This study used adult female Swiss albino mice weighing 22 to 25 g obtained from the animal house of the National Institute of Ophthalmology, Giza, Egypt. Feeding involved standard pellet chow (EL-Nasr Chemical Company, Cairo, Egypt) with free allowance to water. For acclimatization, animals were housed in the same conditions for 1 week prior to the experiment. The experimental work described in this study complies with guidelines for the care and the use of laboratory animals and the ethical principles adopted by the “Research Ethics Committee”, Faculty of Pharmacy, Tanta University (TP/RE/05/22M-0017). To establish tumors in the Swiss Albino mice, an Ehrlich ascites carcinoma (EAC) cell line was supplied from the Pharmacology and Experimental Oncology Unit of the National Cancer Institute (NCI), Cairo University, Egypt. EAC cells can grow in either solid or ascetic forms. The used EAC cells were of mammary origin, where a spontaneous mouse breast carcinoma served as the original tumor. The cells were maintained in ascetic form through intra-peritoneal (I.P.) transplantation of a 2.5 × 10^6^ tumor cell into the peritoneal cavity of the mice and allowed to multiply. This procedure develops ascetic fluid rich in Ehrlich tumor cells after 10 days. The fluid is collected through I.P. puncture using a sterile syringe and diluted with saline before cell counting via a Neubauer Hemocytometer (Spectrum Scientifics, Irvine, CA, USA) [30]. The cells were found to be more than 99% viable by the trypan blue dye exclusion method [31]. The model of solid Ehrlich carcinoma (SEC) was then induced in female Swiss albino mice by injecting 0.2 mL of viable EAC cells (5 × 10^6^/mL) subcutaneously (S.C.) into the right thigh of each mouse and left for 13 days [32,33].

#### 2.8.1. Experimental Design

After the mice developed palpable solid tumors, they were divided randomly into nine groups (6 mice each), as displayed in Table 2. The groups were treated through oral or intratumor administration of TAM suspension, a TAM bilosomes formula (F3), a combined PRO and TAM suspension, or bilosomal dispersion (F3) containing both TAM and PRO. The last group was left as an untreated control group. The administered dose was 10 mg/kg body weight for both drugs. This dose was calculated by taking 40 mg as the human daily dose of both drugs. This dose was divided by the average body weight of humans and converted to a mouse dose through multiplication by the conversion factor [34]. The resulting dose was approximated to 10 mg/kg. The intratumor delivery of anti-cancer drugs was considered a valid therapeutic strategy for the treatment of cancer [35]. The first dose was received after 13 days post-SEC induction (the first day of treatment), while the second dose was received after 19 days post-SEC induction (the seventh day of treatment). Such multiple intratumor doses are reported in the literature [36,37,38]. For those given orally, mice received a daily dose of 10 mg/kg of the tested drugs for two weeks.

#### 2.8.2. Tumor Volume (V) and Percentage of Tumor Growth Inhibition (% TGI) Measurement

Tumor volumes were recorded twice weekly starting from the first day of treatment until the day of mice scarification. Tumor volume was measured before each administration. This was achieved using a Vernier caliper (Trickle Brand, Shanghai, China). The tumor volume was calculated using the following equation [32]:

Tumor volume (mm^3^) = 0.52 AB^2^

where (A) and (B) are the minor and major axis, respectively.

Drug efficiency is expressed as the percentage of tumor growth inhibition (TGI), which was calculated using the following relationship [39]:

% TGI = 100 − (T/C × 100)

where T is the mean relative tumor volume (RTV) of the treated group and C is the RTV of the control group. The RTV of any group was calculated using the following equation [39]:

RTV = Vx/Vi

where Vx is the tumor volume at the end of the experiment (days of scarification) and Vi is the tumor volume on the first day of treatment.

The study was ended after 14 days of drug administration when mice were euthanized, and the tumors were excised and weighed. The tumors were maintained in 10% neutral-buffered formalin (pH 7.4) for subsequent histopathological examination.

#### 2.8.3. Histopathological Examination of Tumor Tissues

Histopathological examination of 5 μm thickness cuts of paraffin sections was conducted after staining with hematoxylin and eosin (H&E). This examination was carried out using a photomicroscope with ×100 magnified images (Olympus BX 51, Olympus America, Melville, NY, USA). Sections were examined for changes, such as tumor giant cells, mitotic figures, and necrosis. The average numbers of mitotic figures and giant cells in 5 high-power fields (HPFs) were calculated and presented as the mean ± SD. Grading of necrosis was performed on a 4-point grading scale in which 0 = absent, 1 = mild, 2 = moderate, 3 = marked, and 4 = diffuse pattern, which represents the greatest extent [40].

### 2.9. Statistical Analysis

All results were statistically analyzed using one-way ANOVA, followed by Tukey’s post-hoc test for pairwise comparisons. Significance was considered when the *p*-value was less than 0.05. These evaluations were carried out using Statistical Package for Social Sciences (SPSS) (SPSS Inc., Chicago, IL, USA) software for Windows, version 26.

## 3. Results and Discussion

### 3.1. Spectrophotometric Determination of Drugs

The UV spectrophotometric assay methods were linear in the range of 5 to 25 µg/mL for TAM and in the range of 5 to 35 µg/mL for PRO. The calibration equation of TAM was Y = 0.0334X + 0.0214, and the calibration equation of PRO was Y = 0.0218X + 0.0127. The assay methods were validated as per the ICH guidelines. The linearity range was enough for drug quantification of the tested samples for drug release studies and entrapment efficiency. The detection and quantification limits for TAM were 0.49 µg/mL and 1.47 µg/mL, respectively. For PRO, the detection limit was 0.69 µg/mL, and the quantification limit was 2.11 µg/mL.

### 3.2. Transmission Electron Microscopy (TEM)

**Figure 2:**
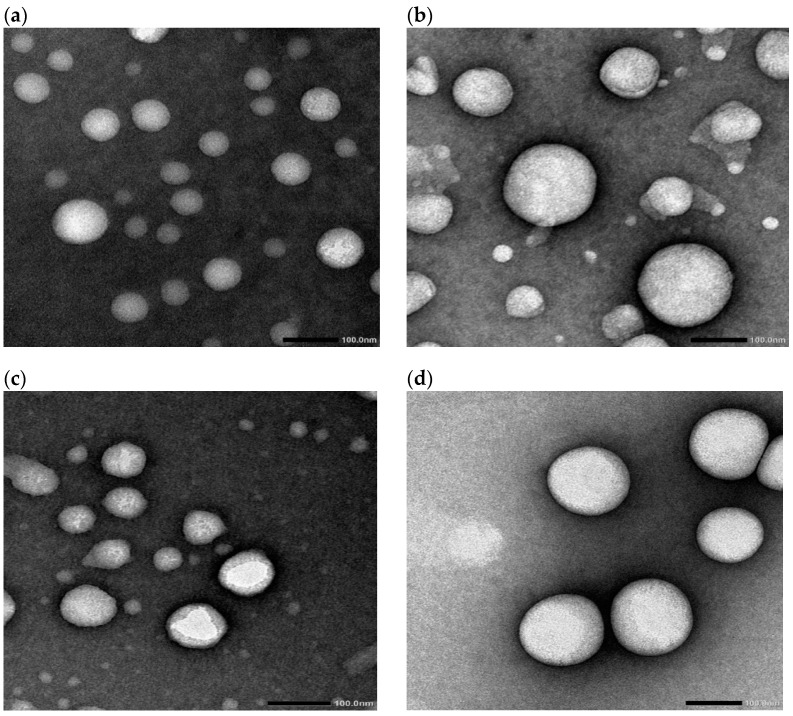
Transmission electron micrographs of F1 (**a**), F2 (**b**), F3 (**c**), and F4 (**d**). Formulation details are presented in Table 1.

Figure 2 shows representative transmission electron micrographs of vesicles containing increasing concentrations of bile salts. The micrographs reflect the spherical nature of the vesicles. The spherical shape was shown irrespective of the composition of the vesicles. This is obvious, as both standard niosomes and bilosomes are spherical vesicular architectures [21,22]. The captured micrographs were utilized to measure the vesicle size, which is presented in Table 3, in comparison with the size values reported through photon correlation spectroscopy (PCS). The TEM-recorded particle size values were 76.8, 110.3, 97.8, and 150.9 nm for F1, F2, F3, and F4, respectively (Table 3). The computed size values of the prepared vesicles correlate with those recorded for niosomes and bilosomes prepared using the same technique [21,22,23].

### 3.3. Particle Size and Zeta Potential

**Figure 3:**
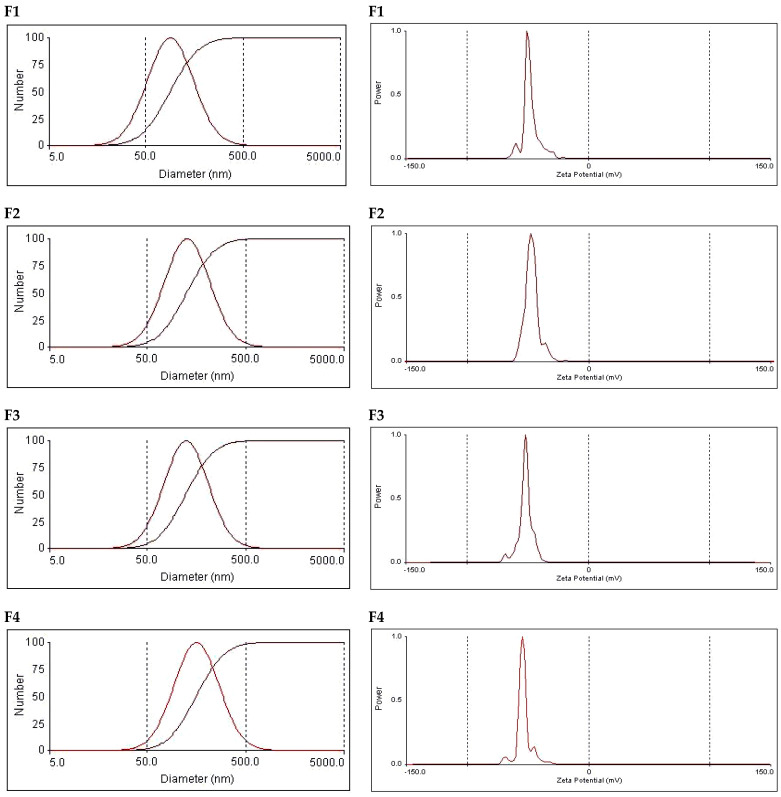
Particle size distribution and zeta potential graphs of the tested vesicular formulations. Formulation details are shown in Table 1.

Particle size values and polydispersity indices (PDIs) were also determined through photon correlation spectroscopy (PCS). The average size and PDI values are presented in Table 3, and the vesicle size distributions are graphically illustrated in Figure 3. The particle size distribution shows a monomodal distribution, with single-peak, bell-shaped curves recorded. This was the case for the tested niosomes and bilosomes. Despite the existence of monomodal distribution, the recorded PDI values were above 0.2 (they ranged from 0.32 to 0.36), reflecting the heterogenicity of the vesicles. These results are expected given the current method of preparation, in which vesicle size reduction involved bath sonication. Bath-sonicated vesicles were previously shown to be heterogenous. Obtaining a similar pattern of size distribution for niosomes and bilosomes is acceptable when taking into consideration the fact that vesicle size distribution depends on the method of preparation [22,23].

The average vesicle size values were 103.9, 145.9, 141.6, and 181.7 nm. These values reflect a gradual increase in vesicle size upon increasing the concentration of bile salt in the formulation. The increase in vesicles size in the presence of bile salts can be due to the charge imparted by the bile salts, which can subsequently result in intermolecular repulsion. This can provide a greater chance of entrapment of a larger volume of aqueous core, which increases the vesicle size. Similar vesicle size values were recorded for bilosomes prepared using the same technique [23]. It is noteworthy that the vesicle size values recorded using the PCS technique were in the same rank compared with those measured using the TEM micrographs, confirming the increased size upon an increased concentration of bile salts. However, the numerical values recorded after PCS measurements differ from the size value of the corresponding formulation measured through TEM. This finding is acceptable when taking into consideration the fact that size measurement based on TEM micrographs depends on the captured field, but PCS provides an average particle size based on the mobility of vesicles in the dispersion. A similar discrepancy has been shown in other studies citing the vesicle size value using the two techniques [23,41].

With respect to the zeta potential, all formulations carried a negative charge, with the numerical values of the zeta potential ranging from −48.4 to −54.9 mV (Table 3 and Figure 3). These values guarantee inter-vesicle repulsion, which imparts physical stability. Standard niosomes (F1) revealed a negative zeta potential, which was attributed to the presence of poly oxygen groups, which can polarize in an aqueous dispersion carrying a small negative charge. Other investigators have attributed the recorded negative zeta potential to the preferential adsorption of hydroxyl ions at the vesicular surface [42]. Similar records of zeta potential were previously reported by other researchers for conventional niosomes comprising Span 60 and cholesterol [25]. Surprisingly, increasing the bile salt concentration did not lead to a significant change in the zeta potential. This may be attributed to the highly negative nature of bile-salt-containing vesicles. Similar data were recorded by other investigators and were considered to be a positive contributing factor to the physical stability of the vesicles [23].

### 3.4. Determination of Entrapment Efficiency

The entrapment efficiency is a determining factor for the feasibility of vesicular carriers for delivery of a given drug. Table 3 presents the calculated entrapment efficiency values of TAM and PRO in the vesicular systems. Regarding TAM, the computed entrapment efficiency values were 87.5%, 97.8%, 95.3%, and 93.5% for standard niosomes (F1) and bilosomes containing bile salts at concentrations of 4, 6, and 8 mg/mL (F2, F3, and F4, respectively). The high entrapment efficiency values correlate with the lipophilic nature of TAM, which dictates its preferential partitioning into the lipidic components of the vesicular lipid bilayer. Localization of lipophilic drugs into the lipid bilayer is documented in the literature [43]. Similar entrapment efficiency values were recorded for other lipophilic drugs [44]. For PRO, lower entrapment efficiency was noted, recorded at 45.3%, 43.6%, 46.8%, and 31.0% for formulations F1, F2, F3, and F4, respectively. The relatively lower entrapment compared to TAM is acceptable when taking into account the hydrophilic nature of PRO. Hydrophilic drugs are expected to be located in the aqueous compartments of the vesicles, and the entrapment efficiency will be mainly dependent on the entrapped volume of the aqueous phase, with a possible contribution from the surface adsorption of the drug on the lipid bilayer [44]. It is noteworthy that bilosomes containing the highest concentration of bile salts showed the lowest entrapment efficiency value of PRO. This is obviously due to the highly fluidizing effect of bile salts, which subsequently allows for the escape of large amounts of the hydrophilic PRO. Low entrapment efficiency was indicated for hydrophilic drugs in earlier literature reports [45,46].

### 3.5. In Vitro Drug Release

**Figure 4:**
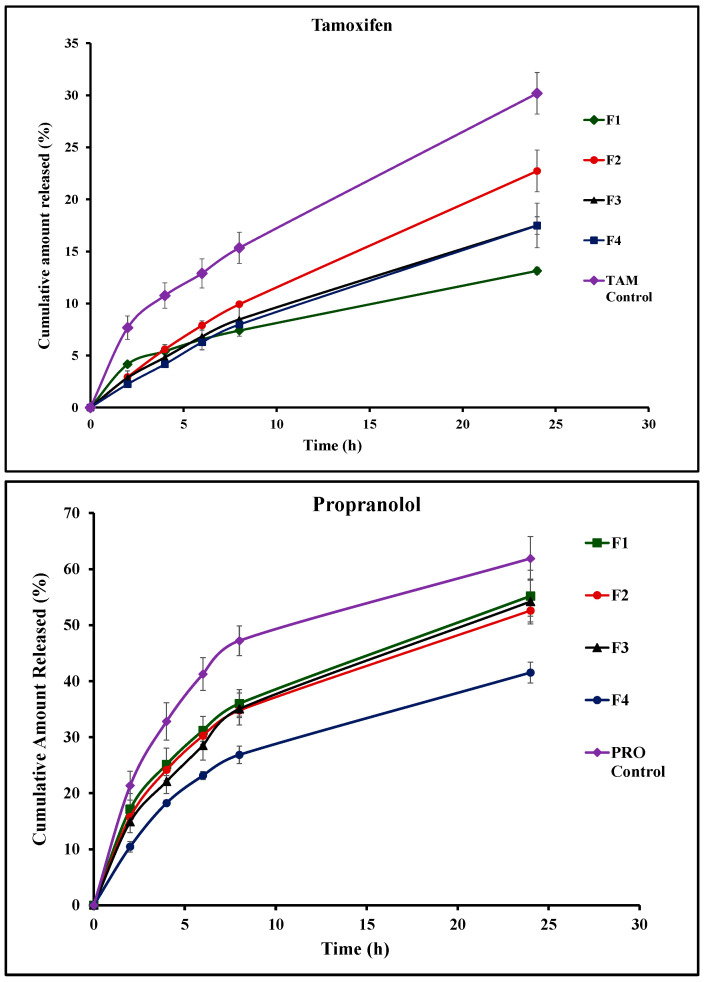
In vitro release profiles of tamoxifen (TAM) and propranolol (PRO) from different vesicular systems and their unprocessed aqueous dispersions (control). Formulation details are presented in Table 1.

In vitro release studies of TAM and PRO depended on the diffusion strategy. This employed Franz diffusion cells with a semipermeable cellulose membrane mounted between the donor and the receptor compartment [47]. Other researchers have previously employed the same technique for monitoring drug release from niosomes and bilosomes [48,49,50]. It is noteworthy that the use of Franz cells for the release studies offers an advantage of a unified effective surface area of the receptor-exposed membrane surface available for drug release throughout the whole release experiment (2.27 cm^2^). The recorded release profiles of TAM and PRO from niosomes (F1), bilosome formulations (F2–F4), and aqueous unprocessed drug dispersion are presented in Figure 4. The calculated release efficiency values are presented in Table 3. The solubility values of TAM and PRO in 0.02 N HCL were previously investigated by other researchers, recorded at 0.2 mg/mL and 225 mg/mL, respectively [51,52]. The initial drug amount loaded in the 2 mL formulation on the dialysis membrane was 2 mg of either TAM or PRO (Table 1). Considering either the TAM or the PRO load in the tested formulations, the employed volume of the release medium in the receptor compartment, and the volume of samples collected through the release study, the sink conditions were ensured throughout the whole study [53,54].

The data of release kinetics are presented in Table 4 after fitting the release profiles to different kinetic models (zero order, first order, and Higuchi). With respect to the release kinetics of the tested vesicular systems, both drugs were liberated based on the Higuchi release kinetic model (Table 4). Such a kinetic model is not expected to exist in the absence of matrix-forming systems. The existence of such a kinetic model in the case of liquid niosomes and bilosomes can be explained on the basis of their multilamellar structure. This architecture allows for rapid drug release from the surface lamellae, with subsequent successive diffusion from the internal ones. This leaching process behaves like a matrix, explaining the feasibility of Higuchi release kinetics for such a fluid system. Similar findings were shown by other researchers, who claimed the same explanation after computing the kinetics of release from vesicles [21].

Release studies of TAM from its unprocessed aqueous dispersion (control) revealed poor TAM release, with the computed release efficiency being 18.4 ± 2.2%. This was expected when taking into consideration the reported poor solubility of TAM [51]. TAM underwent sustained release from the vesicular systems compared to the control aqueous drug dispersion. The rate of drug release depended on the composition of the vesicular system. Standard niosomes showed the slowest release pattern, with the release efficiency being 8.4% (Figure 4 and Table 3). The incorporation of bile salts in the vesicular structure increased the release efficiency (Figure 4 and Table 3). It is noteworthy that upon comparing the overall release profiles using the similarity factor test, a similar release profile is indicated by the computed f2 values, which were more than 50% in all cases. This indicates that the tested concentrations of bile salt provided only a trend of increased drug release efficiency.

For PRO release, a greater release rate was recorded compared with the release of TAM from the corresponding formulation as well as the control drug dispersion. The recorded release efficiency of PRO from its aqueous control dispersion reached 46.3 ± 0.8%. The computed release efficiency values were 38.0%, 36.0%, 36.7%, and 28.0% for F1, F2, F3, and F4, respectively (Table 3). The liberation of the drug from the vesicular systems depends on the physicochemical properties of the drug and its relative affinity to aqueous and lipid components of the vesicles. Lipophilic drugs are expected to undergo better retention and, subsequently, slow release. In contrast, hydrophilic species are expected to diffuse outside of the vesicles at a relatively faster rate compared with the lipophilic drugs. This was reflected in drugs like estradiol and 5-fluorouracil as model lipophilic and polar drugs, respectively [44,45]. This explains the recorded results in the current investigation.

### 3.6. In Vivo Evaluation of Anti-Tumor Activity

**Figure 5:**
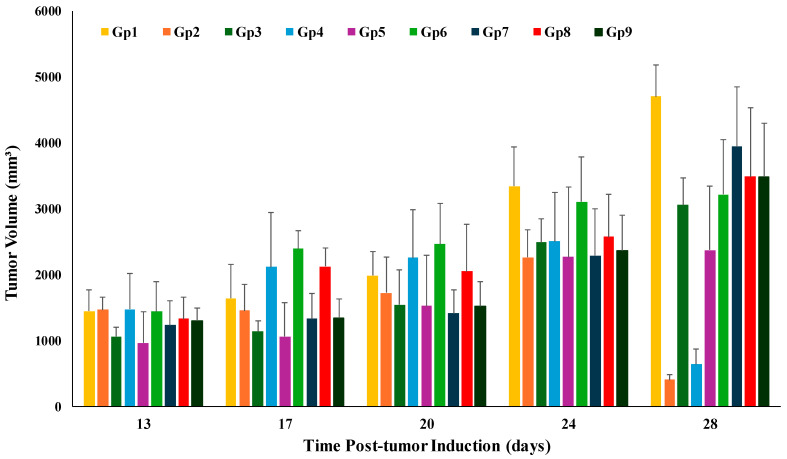
Changes in the tumor volume in the tested animal groups during the experiment’s time course. Gp2, Gp3, Gp4, and Gp5 are the animal groups assigned for oral administration of TAM bilosomes, TAM suspension, TAM/PRO bilosomes, and TAM/PRO suspension, respectively. Gp6, Gp7, Gp8, and Gp9 are the animal groups assigned for intratumor administration of TAM bilosomes, TAM suspension, TAM/PRO bilosomes, and TAM/PRO suspension, respectively. Gp1 represents the untreated animal group.

This study employed the SEC model in the assessment of the anti-tumor activity of the TAM and PRO combination in their suspension and bilosomal formulations using both oral and intratumor routes. This model is a widely used tool in investigating anti-cancer drugs due to its high efficiency in generating neoplastic cells with a long survival time [55]. Moreover, this model is advantageous for tamoxifen, which is used in the treatment of this specific tumor, which allows for extrapolation of the recorded results for future clinical studies with no worry about different resistance patterns among different cell lines. The components utilized in the bilosomal formulation are generally acknowledged as safe. These components have demonstrated perfect biocompatibility and biodegradability [56]. The efficacy of anti-tumor activity was assessed by monitoring the tumor volume. The tumor volume was measured on days 13,17, 20, 24, and 28 after tumor induction, and the data are presented in Figure 5. The percentage of reduction in the tumor volume was calculated relative to the untreated group (the negative control group) to estimate the efficacy of different drug formulations. Tumor volume data were also used to calculate the %TGI, which is presented in Table 2. Further assessment was achieved through histopathological examination, which was used to compute the average number of mitotic figures and giant cells. In addition, the tumor necrosis score was also deduced through histopathology. Statistical analysis results for the comparison between groups and routes of administration are presented in Table 5. There was no significant difference (*p* > 0.05) in the tumor volume values of the tested groups on day 13 (immediately before starting the treatment protocol). This reflects the proper distribution of mice in each group with respect to the starting tumor volume.

**Figure 6:**
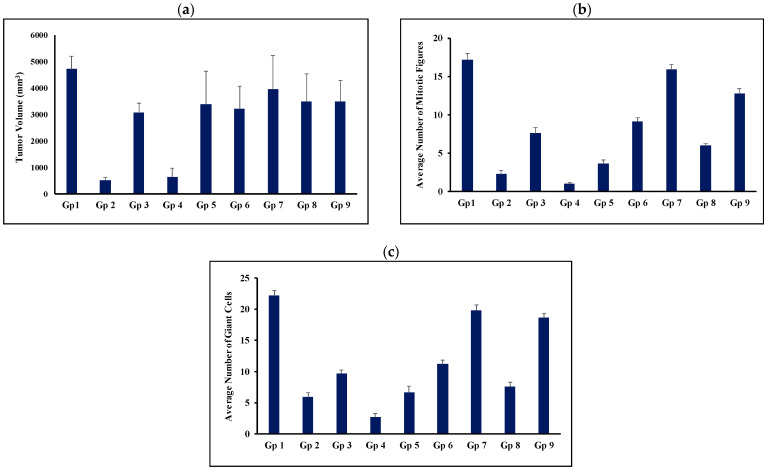
Average tumor volume (**a**), average number of mitotic figures (**b**), and average number of giant tumor cells (**c**) on day 14 post-treatment. Gp2, Gp3, Gp4, and Gp5 are the animal groups assigned for oral administration of TAM bilosomes, TAM suspension, TAM/PRO bilosomes, and TAM/PRO suspension, respectively. Gp6, Gp7, Gp8, and Gp9 are the animal groups assigned for intratumor administration of TAM bilosomes, TAM suspension, TAM/PRO bilosomes, and TAM/PRO suspension, respectively. Gp1 presents the untreated animal group.

With respect to tumor volume, the tumor volumes measured on the 14th day post-treatment (i.e., the 28th day post-tumor induction) for all groups are graphically presented in Figure 6. The untreated group showed a progressive increase in tumor volume from 1448 mm^3^ on day 14 post-induction to 4703 mm^3^ at the end of study, accounting for a 3.25-fold increase in tumor volume. This growth rate correlates with the expectations for tumor growth after proper induction.

Oral administration of TAM suspension reduced the tumor volume compared with the untreated group. This reduction was shown to be statistically significant (Figure 6 and Table 5). However, this was expressed by only 12.7% tumor growth inhibition (Table 2). Co-administration of TAM with propranolol resulted in a significant reduction in tumor volume compared with the untreated group (*p* < 0.05). The %TGI was increased from 12.7% to 18.4% (Table 2). However, the statistical analysis reflected no significant difference between the group treated with TAM suspension alone (Gp 3) and that treated with the TAM/PRO suspension (Gp5) (*p* > 0.05, Table 5).

Oral administration of the bilosomal formulation of TAM resulted in a significant reduction in the tumor volume compared with the untreated control group (*p* < 0.001). This administration inhibited the growth of the tumor by 91.6% (Table 2). Oral administration of TAM/PRO in the bilosomal formulation showed a significant reduction in tumor volume compared with the control (*p* < 0.001), with the %TGI being 86.9% (Table 2). As for the simple suspension formulation, there was no significant difference (*p* > 0.05) between the administration of TAM bilosomes (Gp 2) and TAM/PRO bilosomes (Gp 4) with respect to tumor volume (Figure 6 and Table 5).

Intratumor administration of the TAM suspension did not result in a significant reduction in the tumor volume relative to the untreated control, with the %TGI being only 3%. The %TGI was increased to 19% after the administration of the TAM/PRO suspension (Table 2). However, the variability made this increase statistically non-significant (*p* > 0.05). Intratumor administration of TAM bilosomes or TAM/PRO bilosomes resulted in a significant reduction in tumor volume compared with the untreated control, with the %TGI being 32.4% and 20.8%, respectively (Table 2). Upon comparing the efficiency of route of administration, oral administration was ranked as more efficient than intratumor administration. This was particularly clear after bilosomal administration (Table 5). This finding can be explained on the basis of the administration protocol, as oral administration involved daily dosing, while intratumor administration was performed via once weekly administration of the same dose.

**Figure 7:**
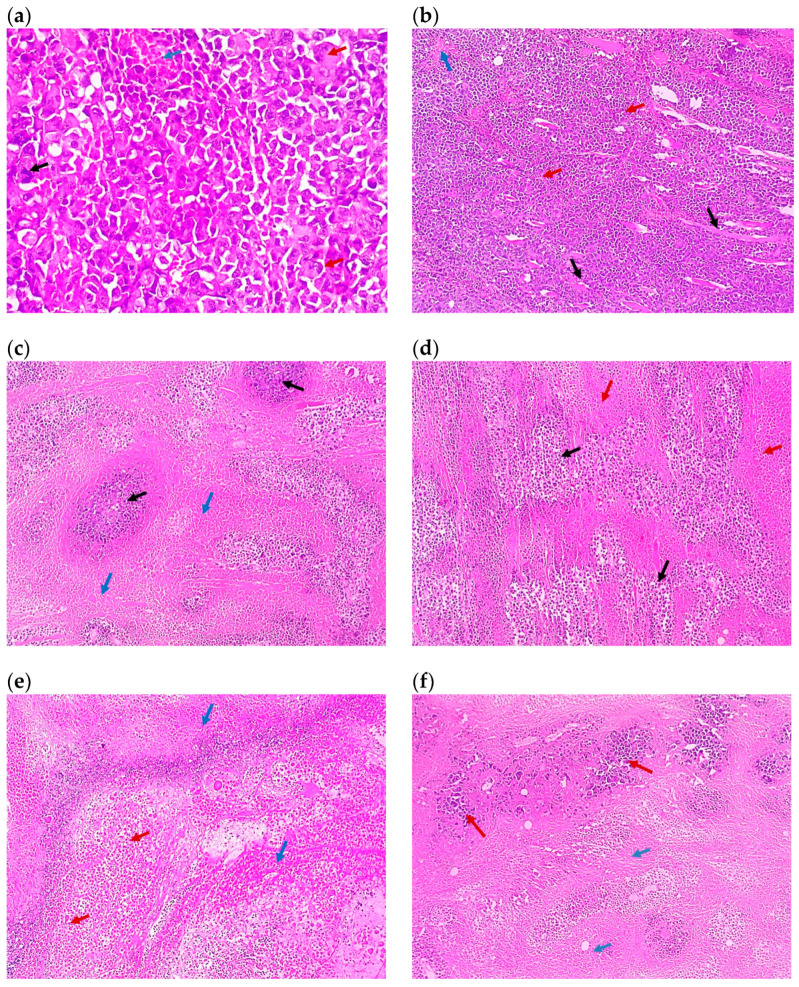

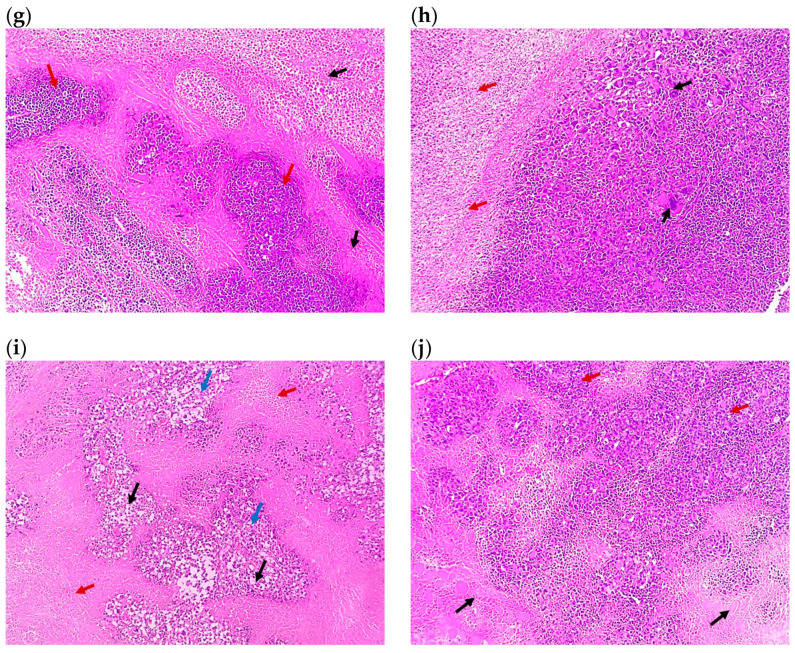
Representative photomicrographs of the tumor sections of (**a**) Group 1, the control (×100), showing solid sheets of malignant cells (red arrows) infiltrating muscle fibers (black arrows) with a focal area of necrosis (blue arrow), (**b**) the control at higher magnification (×400), showing malignant cells exhibiting pleomorphism, hyperchromatisa, and abnormal mitotic figures (black arrow) and tumor giant cells (red arrows) with a focal area of necrosis (blue arrow), (**c**) Group 2 (×100) showing malignant cells (black arrows) surrounded by 80% of tumor necrosis (blue arrows), (**d**) Group 3 (×100), showing malignant cells (black arrows) surrounded by 50% of tumor necrosis (red arrows), (**e**) Group 4 (×100), showing shadows of necrotizing malignant cells (red arrows) surrounded by 95% of tumor necrosis (blue arrows), (**f**) Group 5 (×100), showing malignant cells (red arrows) surrounded by 70% of tumor necrosis (blue arrows), (**g**) Group 6 (×100), showing malignant cells (red arrows) surrounded by 50% of tumor necrosis (black arrows), (**h**) Group 7 (×100), showing malignant cells (black arrows) surrounded by 20% of tumor necrosis (red arrows), (**i**) Group 8 (×100), showing malignant cells (blue arrows) with tumor vacuolar changes (black arrows) surrounded by 60% of tumor necrosis (red arrows), and (**j**) Group 9 (×100), showing malignant cells (red arrows) surrounded by 30% of tumor necrosis (black arrows). All images are captured at magnification power ×100, except (**b**) which is at ×400. Details regarding the different groups are presented in Table 2.

Histopathological examination provided a deeper assessment of the anti-tumor activity, which was monitored further from the histopathological parameters. The tumor necrosis grading revealed the superiority of bilosomal formulation after oral administration. Based on this parameter, the formulations were ranked as TAM/PRO bilosomes (grade 4) > TAM bilosomes (grade 3); TAM/PRO suspension (grade 3) > TAM suspension (grade 2) (Table 2). It is important to highlight that higher grade of tumor necrosis reflects cell death and more intense anti-tumor activity [40]. The potential improvement of tamoxifen anti-tumor efficacy was revealed through this parameter. As for tumor volume, tumor necrosis was lower after intratumor administration compared to oral administration (Table 2 and Figure 7). It is important to highlight the fact that tumor volume data indicated comparable effects for TAM bilosomes and those of TAM/PRO bilosomes, but necrosis grading showed the superiority of TAM/PRO bilosomes. This can be explained on the basis that the TGI value and the necrosis score reflect the drugs’ effects on different aspects of the carcinogenesis process. The necrosis score measures certain forms of cell death, whereas the TGI measures tumor growth and proliferation.

The mitotic figure counts provided another indicator of anti-tumor activity (Figure 6). The mitotic figure count is a good indicator that reflects the magnitude of cell proliferation. Thus, a high count reflects the aggressiveness of the tumor, and a low count reflects the efficiency of the therapy [57,58]. The untreated control group (Gp1) exhibited an average mitotic figure count of 17.16. Oral administration of TAM suspension reduced this count to 7.55. This value was reduced further to 3.56 after combination with PRO, as in the simple dispersion form. Bilosomal encapsulation of TAM or TAM/PRO reduced this value to reach 2.25 and 1, respectively (Figure 6). Statistical evaluation indicated the efficacy of all formulations compared with the untreated group (*p* < 0.05, Table 5). The combination of TAM with PRO showed hastened efficacy, with bilosomal encapsulation being optimum (*p* < 0.05, Table 5). Intratumor administration provided mitotic figure counts of 15.88, 12.76, 9.08, and 6.01 for the TAM suspension, the TAM/PRO suspension, the TAM bilosomes, and the TAM/PRO bilosomes, respectively (Figure 6). This is the same rank as in the case of oral administration but with significantly lower efficiency compared to the oral route (Table 5). Again, regular oral administration was more effective. The reduced mitotic figure count was taken as evidence of a reduced rate of cell division, which subsequently enhanced the anti-tumor activity [57,58].

With respect to the metastatic potential, the giant cell count was adopted. This is used as an indicator of the tendency of spreading. A higher giant cell count reflects a greater chance for metastasis [59]. The giant cell count was 22.18, 9.66, 6.74, 5.89, and 2.7 cells/5HPF for the untreated control, the TAM suspension, the TAM/PRO suspension, the TAM bilosomes, and the TAM/PRO bilosomes, respectively (Figure 6). These results reflect a significant reduction in the giant cell count compared with the untreated control (*p* < 0.05, Table 5). Once again, PRO hastened the efficacy, with the maximum effect being shown in the case of TAM/PRO bilosomes (*p* < 0.05, Table 5). For intratumor injection, the giant cell count was 19.75, 18.57, 11.15, and 7.56 cells/5 HPF for the TAM suspension, the TAM/PRO suspension, the TAM bilosomes, and the TAM/PRO bilosomes, respectively (Figure 6). The oral route was significantly more efficient (*p* < 0.05, Table 5).

The use of propranolol as a potentiating adjuvant with an anti-tumor agent was based on its potential anti-proliferative, anti-angiogenic, anti-lymphangiogenic, pro-apoptotic, and immunomodulating activities [13,60,61]. For example, PRO was able to induce apoptosis in breast cancer cell lines. This was attributed to the disruption of bioenergetics in intact cells. Beta-adrenergic signaling was shown to have a role in the metastasis of breast cancer to the bone [62]. This provides additional benefits for using PRO in conjunction with anti-tumor agents, and it can explain the recorded potentiation in the current study.

The superiority of vesicular carriers in oral drug delivery has been highlighted by other investigators, with alternative mechanisms being hypothesized. These include invasion of the intestine via the lymphatic pathway. This possibility allows for avoidance of pre-systemic metabolism [63,64]. Other mechanisms may rely on the ability of vesicular components to increase the membrane permeability of GIT, with a subsequent increase in drug influx [65]. Additionally, nanocarriers with an average size of less than 200 nm are reported to be passively accumulated in solid tumors via the enhanced permeation and retention effect. Considering bilosomes, incorporation of bile salts in lipid vesicles is believed to increase vesicular membrane flexibility, allowing for better contact with the biological membrane. This characteristic was shown to be important in transdermal delivery [44]. Extrapolation to intestinal absorption requires investigation, but some authors have reported the superiority of bilosomes over traditional vesicles for oral drug delivery [66]. These features can explain the recorded enhancement of anti-tumor activity of tamoxifen alone or in combination with PRO after oral administration of bilosomes compared with the corresponding simple dispersion.

The superiority of oral administration of the tested formulation over intratumor administration may be explained on the basis of different treatment protocols, which involved daily oral dosing versus weekly intratumor administration of the same dose. The results suggest that maintaining a steady plasma level through daily oral dosing is even more efficient than intratumor invasion every week. However, this requires further future clinical investigations.

## 4. Conclusions

Simultaneous encapsulation of tamoxifen (TAM) and propranolol (PRO) was successfully achieved in bile salts with free niosomes and bilosomes. The entrapment efficiency and release depended on the physicochemical properties of the drugs, with the lipophilic tamoxifen recording better entrapment and slower release than the hydrophilic propranolol. The lower entrapment of propranolol is due to its entrapment in the aqueous compartments of the vesicles, which depends on the entrapped volume. Oral administration of tamoxifen reduced the tumor volume, with bilosomal delivery potentiating the efficacy. Co-administration with propranolol hastened the performance of tamoxifen against breast cancer, especially when considering the mitotic figures and the giant cell count. Again, simultaneous bilosomal delivery of TAM and PRO was superior. Daily oral administration was more effective than single intratumor delivery of the corresponding formulation. Maintaining a steady delivery of TAM is required for efficient therapy. Overall, propranolol has high potential as an adjuvant therapy with tamoxifen to increase its efficacy and reduce metastasis in breast cancer therapy, with oral bilosomal delivery being more effective than simple aqueous dispersion. However, this requires further future investigations addressing the stability of the fabricated bilosomal systems. Detailed pharmacokinetics are required in the future for verification. Additionally, the potential of the proposed combination therapy should be evaluated in drug-resistant breast cancer.

## Figures and Tables

**Table 1:** Composition of the prepared formulations.

	Span 60 (mg)	Cholesterol (mg)	Bile Salts (mg)	Ethanol (mL)
F1	600	100	-	1
F2	600	100	100	1
F3	600	100	150	1
F4	600	100	200	1

Tamoxifen and/or propranolol were included at a concentration of 1 mg/mL, and the above composition was enough to prepare 25 mL using distilled water as the main external phase.

**Table 2:** Different mice groups used in the study, indicating the type of drug(s), the dosage form, the route of administration, and the frequency of administration together with the percentage of tumor growth inhibition (%TGI) and the necrosis score.

Group (Gp)	Drug/s	Formulation	Route of Administration	Dose Frequency	TGI (%)	Necrosis Score
Gp 1	-	Vehicle	Oral	Daily	-	-
Gp 2	TAM	Bilosomes	Oral	Daily	91.6	Grade 3
Gp 3	TAM	Suspension	Oral	Daily	12.7	Grade 2
Gp 4	TAM + PRO	Bilosomes	Oral	Daily	86.9	Grade 4
Gp 5	TAM + PRO	Suspension	Oral	Daily	18.4	Grade 3
Gp 6	TAM	Bilosomes	Intratumor	Once/week	32.4	Grade 2
Gp 7	TAM	Suspension	Intratumor	Once/week	3.0	Grade 1
Gp 8	TAM + PRO	Bilosomes	Intratumor	Once/week	20.8	Grade 2
Gp 9	TAM + PRO	Suspension	Intratumor	Once/week	19.0	Grade 1

Grading of the extent of necrosis was performed on 4-point grading scales, as follows: 0 for absence, 1 for mild, 2 for moderate, 3 for marked, and, finally, 4 for the diffuse pattern, which is the greatest extent.

**Table 3:** Characterization parameters of the tested niosomal and bilosomal systems. The composition of the formulations is presented in Table 1.

Formulation	EE (%)		Particle Size (nm)		PDI	ZP (mv)	RE (%)	
	TAM	PRO	TEM	PCS			TAM	PRO
F1	87.5 ± 2.4	45.3 ± 4.4	76.8 ± 28.5	103.9 ± 45.5	0.36	−51.3 ± 7.4	8.4 ±0.1	38.0 ± 2.8
F2	97.8 ± 0.2	43.6 ± 1.6	110.3 ± 36.5	145.9 ± 55.1	0.34	−48.4 ± 3.0	11.7 ± 1.8	36.0 ± 3.2
F3	95.3 ± 0.9	46.8 ± 1.8	97.8 ± 32.0	141.6 ± 58.8	0.32	−51.7 ± 2.9	9.4 ± 1.5	36.7 ± 3.3
F4	93.5 ± 0.4	31.0 ± 0.7	150.9 ± 33.2	181.7 ± 56.8	0.33	−54.9 ± 4.0	9.8 ± 0.5	28.0 ± 1.3

EE is the entrapment efficiency, ZP is the zeta potential, RE is the release efficiency, TEM is transmission electron microscopy, PDI is the polydispersity index, and PCS is photon correlation spectroscopy.

**Table 4:** Correlation coefficient values (R^2^) recorded after fitting the release data of tamoxifen and propranolol from vesicular systems. The composition of the vesicles is presented in Table 1.

Formulation	Release Kinetics (R^2^ Value)					
	Tamoxifen			Propranolol		
	Zero	First	Higuchi	Zero	First	Higuchi
F1	0.988	0.923	0.997	0.723	0.646	0.838
F2	0.992	0.878	0.995	0.897	0.769	0.965
F3	0.984	0.838	0.999	0.822	0.700	0.912
F4	0.986	0.825	0.997	0.866	0.742	0.946

**Table 5:** Statistical analysis (*p* value) for comparison of the effect of different formulations with respect to various parameters on day 14 after oral administration and intratumor administration.

**Tumor Volume (mm^3^)**									
	**Gp 1**	**Gp 2**	**Gp 3**	**Gp 4**	**Gp 5**	**Gp 6**	**Gp 7**	**Gp 8**	**Gp 9**
Gp 1	-	0.000	0.005	0.000	0.031	0.124	0.699	0.269	0.264
Gp 2	0.000	-	0.000	0.998	0.000	0.001	0.000	0.000	0.000
Gp 3	0.005	0.000	-	0.000	0.919	1.000	0.729	0.993	0.993
Gp 4	0.000	0.998	0.000	-	0.000	0.001	0.000	0.000	0.000
Gp 5	0.031	0.000	0.919	0.000	-	1.000	0.969	1.000	1.000
Gp 6	0.124	0.001	1.000	0.001	1.000	-	0.731	0.991	0.991
Gp 7	0.699	0.000	0.729	0.000	0.969	0.731	-	0.933	0.930
Gp 8	0.269	0.000	0.993	0.000	1.000	0.991	0.933	-	1.00
Gp 9	0.264	0.000	0.993	0.000	1.000	0.991	0.930	1.00	-
**Mitotic Figures**									
	**Gp 1**	**Gp 2**	**Gp 3**	**Gp 4**	**Gp 5**	**Gp 6**	**Gp 7**	**Gp 8**	**Gp 9**
Gp 1	-	0.000	0.000	0.000	0.000	0.000	0.013	0.000	0.000
Gp 2	0.000	-	0.000	0.015	0.010	0.000	0.000	0.000	0.000
Gp 3	0.000	0.000	-	0.000	0.000	0.001	0.000	0.002	0.000
Gp 4	0.000	0.015	0.000	-	0.000	0.000	0.000	0.000	0.000
Gp 5	0.000	0.010	0.000	0.000	-	0.000	0.000	0.000	0.000
Gp 6	0.000	0.000	0.001	0.000	0.000	-	0.000	0.000	0.000
Gp 7	0.013	0.000	0.000	0.000	0.000	0.000	-	0.000	0.000
Gp 8	0.000	0.000	0.002	0.000	0.000	0.000	0.000	-	0.000
Gp 9	0.000	0.000	0.000	0.000	0.000	0.000	0.000	0.000	-
**Giant Cell Count**									
	**Gp 1**	**Gp 2**	**Gp 3**	**Gp 4**	**Gp 5**	**Gp 6**	**Gp 7**	**Gp 8**	**Gp 9**
Gp 1	-	0.000	0.000	0.000	0.000	0.000	0.000	0.000	0.000
Gp 2	0.000	-	0.000	0.000	0.335	0.000	0.000	0.000	0.015
Gp 3	0.000	0.000	-	0.000	0.000	0.040	0.000	0.001	0.000
Gp 4	0.000	0.000	0.000	-	0.000	0.000	0.000	0.000	0.000
Gp 5	0.000	0.335	0.000	0.000	-	0.000	0.000	0.611	0.000
Gp 6	0.000	0.000	0.040	0.000	0.000	-	0.000	0.000	0.000
Gp 7	0.000	0.000	0.000	0.000	0.000	0.000	-	0.000	0.131
Gp 8	0.000	0.000	0.001	0.000	0.611	0.000	0.000	-	0.000
Gp 9	0.000	0.015	0.000	0.000	0.000	0.000	0.131	0.000	-

Gp2, Gp3, Gp4, and Gp5 are the animal groups assigned for oral administration of TAM bilosomes, TAM suspension, TAM/PRO bilosomes, and TAM/PRO suspension, respectively. Gp6, Gp7, Gp8, and Gp9 are the animal groups assigned for intratumor administration of TAM bilosomes, TAM suspension, TAM/PRO bilosomes, and TAM/PRO suspension, respectively. Gp1 presents the untreated animal group. Details of each animal group (classified according to the administered drug(s) and its dosage form) are presented in Table 2.

## Data Availability

The data presented in this study are available upon request from the corresponding author.
